# Acute Effects of an Afterschool Running and Reading Program on Executive Functioning in Children: An Exploratory Study

**DOI:** 10.3389/fpubh.2020.593916

**Published:** 2020-11-19

**Authors:** Jeffrey D. Graham, Emily Bremer, Chloe Bedard, Pallavi Dutta, Michelle Ogrodnik, John Cairney

**Affiliations:** ^1^Department of Family Medicine, McMaster University, Hamilton, ON, Canada; ^2^Faculty of Kinesiology and Physical Education, University of Toronto, Toronto, ON, Canada; ^3^Department of Health Research Methods, Evidence, and Impact, McMaster University, Hamilton, ON, Canada; ^4^Department of Kinesiology, McMaster University, Hamilton, ON, Canada; ^5^School of Human Movement and Nutrition Sciences, University of Queensland, Brisbane, QLD, Australia

**Keywords:** cognition, youth, physical activity, exercise, school

## Abstract

**Objective:** Emerging research within school settings suggests acute forms of physical activity and exercise lead to improvements in executive functioning among children. However, research pertaining to these effects within the afterschool setting remains limited. The primary purpose of this study was to investigate the acute effects of a community-based afterschool running and reading program on executive functioning in 8 to 12-year-old children.

**Method:** Fifty participants were initially recruited to participate in this study. However, due to the COVID-19 pandemic, data collection was terminated prematurely which resulted in a sample size of 15 participants. Participants (*N* = 10) from School 1 completed two batteries of executive function assessments (i.e., inhibition, switching, and updating) separated by 15-min of running or 15-min of sedentary reading. Whereas, only 5 participants from School 2 completed assessments of executive functioning prior to and following the running portion of the program (due to the early termination of data collection).

**Results:** Overall, executive function scores improved across each assessment following the running condition when compared to the reading condition (School 1). Inhibition scores significantly improved, and these effects were very large (School 1). Across both schools, improvements in executive functioning following the running portion of the program ranged from small-large in effect size.

**Conclusion:** Findings from the present study provide initial evidence for the acute effects of a community-based afterschool running and reading program on executive functioning in children. Future research with larger samples in afterschool settings is recommended to replicate this preliminary work.

## Introduction

Regular participation in physical activity is associated with several adaptive physical, cognitive, and mental health outcomes among children and youth (1, 2). Research also suggests acute participation in various forms of physical activity and exercise can positively affect cognition (i.e., long-term memory and aspects of executive functioning) associated with learning and academic performance (3). However, physical inactivity is a global problem among all age groups and particularly among children and youth (4). In Canada, less than one-third of children are meeting daily physical activity recommendations (5). Although several intervention efforts have been pursued to increase physical activity levels among children and youth, the evidence pertaining to the effectiveness of these interventions remains mixed (6). As such, there has been a recent call for a “comprehensive public health initiative" as a means to address physical inactivity levels among children and adolescents that includes evidence-based strategies applied across several societal sectors (7).

The school setting is one sector that has gained substantial interest for physical activity interventions as children and youth spend most of their day sitting during school hours. Despite the short- and long-term success demonstrated by both classroom- and physical-education based physical activity interventions on various physical, cognitive, and academic outcomes (8–10), evidence suggests these interventions have a small impact on physical activity outside of regular school hours (11). Considering these findings, recent efforts have begun to investigate the effects of physical activity interventions within the afterschool setting as another avenue to positively impact the physical and mental health of children during out-of-school hours. However, in comparison to school-based physical activity interventions and acute studies, the total number of studies conducted and the evidence regarding the efficacy of physical activity interventions (12, 13) and acute studies [e.g., (14, 15)] within the afterschool setting remains limited.

Despite emerging interest in evaluating afterschool physical activity programs, most of the research has examined aspects of physical activity participation and physical health as outcome measures (12, 13). As the afterschool setting has also been used to promote learning (16), it would seem beneficial to take advantage of the potential acute effects of physical activity on aspects of cognition (i.e., executive functioning) related to learning. However, we are unaware of previous research examining the acute effects of physical activity on executive functioning within the afterschool setting.

The overarching objective of this study was to investigate the acute effects of a community-based afterschool running and reading program on executive functioning in 8 to 12-year-old children. Specifically, we wanted to ascertain whether the running portion of the afterschool program, which consisted of 15-min of running laps around the school gym or a predetermined track throughout the school, led to acute (or immediate) increases in executive functioning when compared to a sedentary control condition. Consistent with the literature reviewed above, we hypothesized the running portion of the afterschool program would lead to improvements in executive functioning when compared to the sedentary reading portion of the program.

## Methods

### Participants and Design

Participants were 15 grade 3–6 children (*n* = 3 females; *M*_*age*_ = *8.99* ± 1.09) who were part of a larger intervention study investigating the effects of a community-based afterschool running and reading program on aspects of physical fitness, executive functioning, and psychosocial well-being over the course the 2019–2020 school year. The larger study was completed in partnership with the Start2Finish organization (https://www.start2finishonline.org/) and the Dufferin-Peel Catholic District School Board. Two schools were chosen to participate by the Start2Finish organization.

The present study is an examination of the acute effects of the running portion of the program on executive functioning when compared to the sedentary reading portion of the program. Due to requests from the Start2Finish organization and the school board, all of the participants who had consent to participate in the larger study and were enrolled in the afterschool program (i.e., excluding the control participants who were not enrolled in the after school program) were also invited to participate in the acute portion of the study. This resulted in 50 participants from the larger intervention study who agreed to participate in the acute study. However, we conducted a formal sample size calculation [using G^*^Power 3.1.9.2; (17)] prior to data collection based on medium effect sizes (i.e., *f* = 0.25) for the acute effects of physical activity on aspects of executive functioning (i.e., inhibition, switching, and updating) that were derived from the Pontifex et al. (18) review, with power = 0.80 and α = 0.05, which indicated a sample of *N* = 24 was sufficient for analysis.

Nevertheless, due to the COVID-19 pandemic, only 10 participants (School 1) completed the full protocol (i.e., assessments of executive function prior to and following both the running and reading portions of the afterschool program) and 5 participants (School 2) completed assessments of executive functioning prior to and following the running portion of the program. Therefore, randomization at the group level (i.e., counterbalancing the order of the experimental and control conditions) did not occur among the 10 participants who completed the full protocol. In addition, 5 participants only completed the pre- and post-assessments of executive function prior to and following the running portion which limits a true comparison to the control condition among these participants.

The study was approved by the University of Toronto Research Ethics Board and the Dufferin-Peel Catholic District School Board. Parents provided informed written consent and students provided informed written assent before participation in the study.

### Procedure

**Figure 1:**
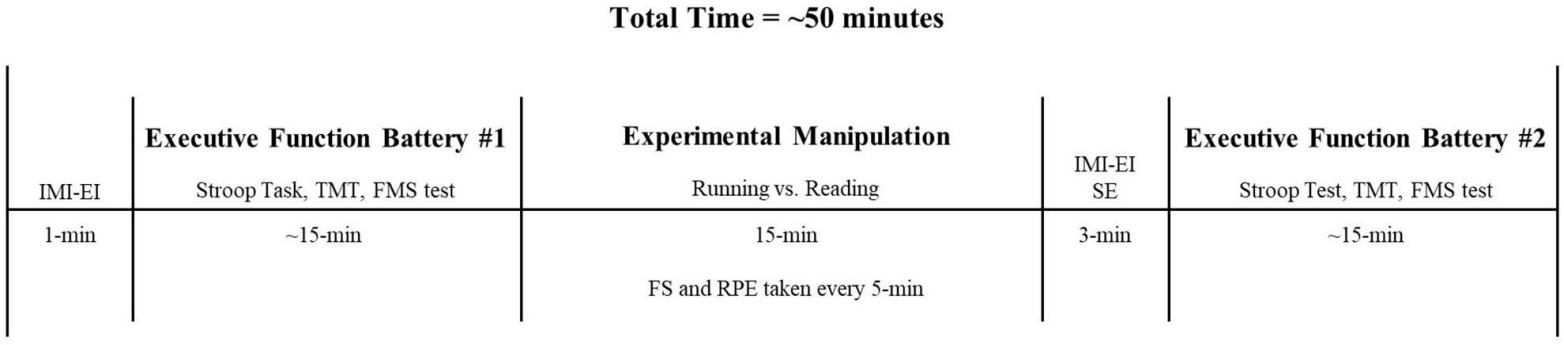
Experimental protocol. IMI-EI, intrinsic motivation inventory–effort and importance subscale; TMT, Trail Making Test; FMS, Forward Memory Span; FS, Feeling Scale; RPE, ratings of perceived physical exertion; SE, self-efficacy.

The data was collected within the first 45-min of the afterschool program. After attendance was taken, participants were accompanied by a trained research assistant to a quiet room within the school to complete the first battery of executive function assessments (see the primary outcome measures section below) while seated at a table. For the running (i.e., experimental) condition, participants were then walked back to the school gym, by the research assistant, at which point they engaged in 15-min of running (see the Start2Finish afterschool program section below) with their peers in the afterschool program. For the reading (i.e., control) condition, participants remained seated at the table and read a grade appropriate book (chosen by the participant) for 15-min. Following the experimental manipulation, participants completed the second battery of executive function assessments in the same quiet room within the school. Upon completion of the second battery of executive function tests, participants were walked back to the afterschool program. For an overview of the study protocol see Figure 1.

### Start2Finish Running and Reading Club

The Start2Finish Running & Reading Club is an inclusive afterschool program offered to low-income schools and students at no-cost. In brief, the program consists of a running portion followed by a reading portion (for more information see https://www.start2finishonline.org/ running-and-reading-clubs). For the purpose of this study, the running portion consisted of 15-min whereby children ran laps around a gym (School 1) or ran laps throughout a predetermined track setup throughout the gym and school halls (School 2).

### Primary Outcome Measures

#### Executive Functioning

Three tasks were chosen to assess the three core executive functions (i.e., inhibition, switching, and updating) that have been commonly used in previous research to assess executive functioning in general (19) as well as to assess the effects of acute exercise on executive functioning in children and youth (18, 20). At each assessment, these tasks were administered in the same order beginning with the Stroop task, followed by the Trail Making Test, and then the Forward Working Memory test.

#### Inhibition

Inhibition was assessed using the congruent and incongruent versions of the Stroop task (21). Participants performed the congruent version for 30-s followed by the incongruent version for 3-min. Both versions consisted of lists of words printed on laminated sheets of paper. In the congruent version, the words and the print ink color were matched (e.g., ink color was *blue* and the word text read *blue*), and participants were asked to read the word aloud (i.e., blue). In the incongruent version, the words and print ink color were mismatched (e.g., ink color was *green* and the word text read yellow), and participants were asked to say aloud the ink color they saw (i.e., green) without reading the word text. In both versions, children were asked to try to respond as fast and accurately as possible. If an error was made it was recorded by the research assistant. A total Stroop task performance score (i.e., Stroop accuracy) was computed by subtracting the number of errors made on each version from the number of words completed on each version, and then summing those values (i.e., Stroop task performance score = [congruent words completed – congruent errors made] + [incongruent words completed – incongruent errors made]). This calculation was conducted separately for the pre- and post-test assessments. Higher scores indicate better performance on the Stroop task.

#### Switching

Switching was assessed using the Trail Making Test [TMT; (22)] as it is a valid and appropriate measure for children (23). The TMT consists of two parts, Part A and Part B. Part A requires participants to connect number sequences, whereas Part B requires participants to alternate between number and letter sequences. In both versions, participants are required to connect the sequences in order, as fast and accurately as possible, without lifting their pencil or turning the paper. If an error was committed (i.e., connected the wrong sequence) or a pencil lift was made, it was recorded by the research assistant under “total errors.” A total TMT performance score was computed by adding the total errors committed to the time (in seconds) it took the participants to complete each version, and then summing those values (i.e., TMT performance score = [total time Part A + total errors Part A] + [total time Part B + total errors Part B). Lower scores indicate better performance on the TMT.

#### Updating

Updating was assessed using the Forward Memory Span (FMS) test from the Leiter International Performance Scale−3rd Edition as it is a valid and appropriate measure for children (24). The FMS test is a non-verbal assessment whereby the participant is presented with pictures of objects shown in a grid pattern (e.g., a 3 × 3 grid). First, the researcher points to multiple pictures (e.g., 4 pictures) in a predetermined order and then the participant is required to copy the same order. The grid pattern and number of pictures gradually increases as the test progresses. An error is recorded if the participant points to any of the pictures in the incorrect order. The test is terminated when six errors are committed or if the participant advances to the last sequence. A total FMS performance score was computed by summing the correct number of sequences performed, with a maximum score of 28. Higher scores indicate better performance on the FMS test.

### Secondary Outcome Measures

While the acute effects of physical activity and exercise on executive functioning are well-established, intermediary processes contributing to these effects remain relatively elusive. As such, we wanted to ascertain whether changes in various psychological perceptions known to result from participation in physical activity and that are directly associated with changes in behavior [e.g., (25–29)], may have also been affected by participation in the running portion of the program.

#### Motivation

Prior to completing each executive function battery, the effort and importance subscale from the Intrinsic Motivation Inventory (30) was used to assess motivation for performing the executive function tests. The subscale contains 5-items that are rated on a scale ranging from 1 (*Not at all true*) to 7 (*Very true*). Each item was prefaced with the following stem “*For the brain games I'm about to do*.” An example item is: “*I am going to try very hard to do well at these brain games*.” A total motivation score was computed by averaging the 5-items at each assessment. Internal consistency at each assessment was good (α's > 0.82).

#### Task Self-Efficacy

Prior to completing the second executive function battery, self-efficacy to perform the executive function tests was assessed using a four-item scale adhering to recommendations by Bandura (31) for assessing self-efficacy. Each item was prefaced with the stem “*For the brain games I am about to do, I am confident I can perform…*”. The individual items represented gradations of performance that were relative to the participant's performance on the first battery of executive function tests. They were (1) “*Almost as good as last time*,” (2) “*As good as last time*,” (3) “*A little better than last time*,” and (4) “*A lot better than last time*.” Participants rated their confidence (i.e., self-efficacy) for each item using an 11-point scale ranging from 0 (*Not at all Confident*) to 10 (*Completely Confident*). The task self-efficacy score was calculated by averaging the items. Internal consistency of the scale was good (α = 0.81).

#### Affect

The Feeling Scale (32) was used to measure affect at 5-, 10-, and 15-min during the experimental manipulations. Participants were asked to rate their current feeling state using an 11-point bipolar single item scale ranging from −5 (*Very Bad*) to +5 (*Very Good*). A total affect score was calculated by averaging the three values collected during the experimental manipulation.

#### Ratings of Perceived Exertion

Participants rated their perceived physical exertion (RPE) at 5-, 10-, and 15-min during the experimental manipulations using Borg's (33) CR-10 scale. Participants were instructed to rate their perception of physical exertion from 0 (*no exertion at all*) to 12 (*absolute maximum*), with 10 (*extremely strong*) representing the highest physical exertion they had ever experienced. A total RPE score was calculated by averaging the three values collected during the experimental manipulation.

### Data Analysis

All statistical analyses were conducted using SPSS 25 (34). Descriptive statistics were computed for all study variables. For the 10 participants who completed the full experimental protocol (School 1), separate 2 (running vs. reading) × 2 (pre- vs. post-experimental manipulation) repeated measures ANOVAs were computed to assess differences in means between conditions for the primary outcome measures and motivation. Separate one-way ANOVAs were computed to assess differences in means between conditions for task self-efficacy, affect, and RPE. Significant interactions were decomposed and evaluated using paired *t*-tests by comparing group means. For the 5 participants who only completed study measures pre- and post-running (School 2), paired *t*-tests were computed to assess differences in means for the primary outcome measures and motivation. As task self-efficacy, affect, and RPE are represented by a single score, descriptive statistics (means and SDs) are presented. Effect sizes for the one-way ANOVAs are reported as Cohen's *d* (35) and the values for small, medium and large are 0.20, 0.50, and 0.80, respectively. Effect sizes for the repeated measures ANOVAs are reported as partial eta squared (*np*^2^) and the values for small, medium, and large are 0.01, 0.06, and 0.14, respectively.

## Results

Descriptive statistics, ANOVA summaries, *t*-test summaries, and effect sizes for the primary and secondary outcome measures are shown, by group, in Table 1 (School 1) and Table 2 (School 2).

**Table 1:** Primary and secondary outcomes for participants from School 1 (*n* = 10).

**Variable**	**Reading**		**Running**				
	**Pre**	**Post**	**Pre**	**Post**	***F***	***p***	**Effect size**
	***M* (*SD*)**	***M* (*SD*)**	***M* (*SD*)**	***M* (*SD*)**			
Stroop task	153.20 (28.84)	148.10 (34.30)	131.10 (33.79)	153.40 (28.76)	23.62	0.001	0.57^a^
Trail Making Test	195.14 (38.60)	198.99 (46.17)	228.82 (47.97)	206.97 (59.80)	2.41	0.14	0.12^a^
Forward Memory Span	21.20 (2.82)	20.50 (1.43)	19.70 (2.00)	20.40 (2.07)	1.91	0.18	0.10^a^
Motivation	6.06 (1.30)	6.06 (1.35)	5.78 (1.22)	5.98 (1.44)	0.88	0.36	0.05^a^
Task self-efficacy	–	5.68 (3.05)	–	8.10 (2.04)	4.36	0.05	0.93^b^
Affect	–	3.47 (1.10)	–	3.60 (1.42)	0.06	0.82	0.10^b^
RPE	–	3.43 (2.71)	–	7.63 (2.19)	14.51	0.001	1.71^b^

*M, mean; SD, standard deviation; RPE, ratings of perceived physical exertion*.

a *Partial eta squared* ($\eta_{\text{p}}^{2}$).

b *Cohen's d*.

**Table 2:** Primary and secondary outcomes for participants from School 2 (*n* = 5).

**Variable**	**Pre-running (*M SD*)**	**Post-running M (*SD*)**	***t***	***p***	***d***
Stroop Task	150.40 (29.77)	163.60 (36.06)	−1.37	0.24	0.40
Trail Making Test	160.12 (50.79)	148.35 (49.56)	1.40	0.26	0.23
Forward Memory Span	20.80 (0.45)	21.40 (1.95)	−0.74	0.50	0.42
Motivation	5.00 (1.03)	5.20 (1.47)	−0.82	0.46	0.16
Task Self-Efficacy	–	5.56 (3.29)	–	–	–
Affect	–	3.11 (2.46)	–	–	–
RPE	–	7.89 (4.76)	–	–	–

*M, mean; SD, standard deviation; RPE, ratings of perceived physical exertion; d, Cohen's d*.

### Primary Analyses

#### Inhibition

Results of the 2 × 2 repeated measures ANOVA for the Stroop task performance scores from School 1 revealed a significant main effect for time (*p* = 0.007, $\eta_{\text{p}}^{2}$ = 0.34) and a significant time by condition interaction (*p* < 0.001, $\eta_{\text{p}}^{2}$ = 0.57). Specifically, as seen in Table 1, Stroop task performance scores significantly increased from pre- to-post-test in the running condition (*p* < 0.001, *d* = 0.74) whereas they decreased slightly in the reading condition (*p* = 0.31, *d* = 0.16). Results of the paired *t*-test for the Stroop task performance scores from School 2 revealed no significant effects (*p* = 0.24, *d* = 0.40).

#### Switching

Results of the 2 x 2 repeated measures ANOVA for the TMT performance scores from School 1 revealed no significant findings for the main effect for time (*p* = 0.29, $\eta_{\text{p}}^{2}$ = 0.07) and the time by condition interaction (*p* =0.14, $\eta_{\text{p}}^{2}$ = 0.12). Results of the paired *t*-test for the TMT performance scores from School 2 revealed no significant effects (*p* = 0.26, *d* = 0.23).

#### Updating

Results of the 2 × 2 repeated measures ANOVA for the FMS test performance scores from School 1 revealed no significant findings for the main effect for time (*p* = 1.00, $\eta_{\text{p}}^{2}$ = 0.00) and the time by condition interaction (*p* = 0.18, $\eta_{\text{p}}^{2}$ = 0.10). Results of the paired *t*-test for the FMS test performance scores from School 2 revealed no significant effects (*p* = 0.50, *d* = 0.42).

### Secondary Analyses

#### Motivation

Results of the 2 x 2 repeated measures ANOVA for motivation scores from School 1 revealed no significant findings for the main effect for time (*p* = 0.36, $\eta_{\text{p}}^{2}$ = 0.05) and the time by condition interaction (*p* =0.36, $\eta_{\text{p}}^{2}$ = 0.05). Results of the paired *t*-test for motivation scores from School 2 revealed no significant effects (*p* = 0.46, *d* = 0.16).

#### Task Self-Efficacy

Results of the one-way ANOVA for task self-efficacy scores from School 1 approached significance (*p* = 0.05, *d* = 0.93). The task self-efficacy score from School 2 can be seen in Table 2.

#### Affect

Results of the one-way ANOVA for affect scores from School 1 revealed no significant findings (*p* = 0.82, *d* = 0.10). The mean affect score from School 2 can be seen in Table 2.

#### RPE

Results of the one-way ANOVA for RPE scores from School 1 revealed significant differences (*p* = 0.001, *d* = 1.71). The mean RPE score from School 2 can be seen in Table 2.

## Discussion

The present study investigated the acute effects of a community-based afterschool running and reading program on executive functioning in 8 to 12-year-old children. It was hypothesized that the physical activity (i.e., running) portion of the afterschool program would lead to improvements in executive functioning when compared to the sedentary reading portion of the program. Although the COVID-19 pandemic terminated data collection early limiting our sample size, we found improvements across all the executive function measures following the running portion of the afterschool program. Specifically, when compared to pre-test scores, post-test executive function scores showed improvements in the hypothesized direction across each measure and these improvements ranged from small to very large-sized effects (see Tables 1, 2).

Fortunately, we were able to collect complete pre- and post-running and reading data from 10 participants (School 1) prior to data collection ceasing, allowing us to conduct within-subject pre-test post-test analyses which is considered the gold standard for testing acute exercise effects on executive functioning (18). Results showed significant and very large effects for improvements in inhibition following the running portion in comparison to the reading portion. Although the findings for task switching and updating were not statistically significant, we found large interaction effects in the expected directions. These findings are encouraging and novel for several reasons. For instance, as far as we are aware, this is the first study to investigate the acute effects of an afterschool physical activity program on executive functioning and results suggest 15-min of running laps can lead to improvements in aspects of executive functioning associated with learning and academic performance. Second, and of particular importance, is the application of these findings within the current program and other afterschool (or other school) settings that seek to utilize the acute positive effects of physical activity on executive functioning and aspects of academic performance such as literacy and numeracy skills. That is, following the running portion of the program in this afterschool program children participate in a sedentary reading portion that works to develop the children's literacy skills. Findings from the present study suggest children's inhibition abilities are enhanced (at least temporarily) during the reading portion which should help them focus, remain still, and manage distractions to aid in the learning process during the reading portion of the program. Task switching and updating abilities were also enhanced following the running portion which should aid in learning by switching back and forth between concepts while keeping alternating concepts within working memory; these skills are essential for learning and are regularly called upon in a typical school day. Finally, as previous research suggests regular engagement in physical activity can lead to improvements in cognition and brain health over time (36), our preliminary findings highlight the potential ability of the program to improve executive functioning over time in general and among low-income students who may benefit the most from an afterschool running and reading program.

Findings from our secondary outcome measures from participants at School 1 also showed small to large-sized effects following the running portion when compared to the reading portion. That is, task self-efficacy scores approached conventional levels of significance (*p* = 0.05) and were large in effect size. As ample research suggests self-efficacy is a strong and reliable predictor of performance and behavior including among children and youth (25), findings from the present study suggest children were more confident in their abilities to perform the second executive function battery following the running portion (when compared to the reading portion) and this confidence may have translated over to actual performance in various ways and likely due to a psychophysiological response. For instance, increases in affective valence are often found following acute moderately intense physical activity [e.g., (27)], such as jogging or running laps. Based on self-efficacy theory (25), increases in affect lead to increases in self-efficacy alongside several neurophysiological processes [see (25), “*Biological Effects of Perceived Self-Efficacy*,” p. 262–278] that may ultimately account for improvements in executive functioning through the upregulation of cerebral blood flow or other brain activation patterns associated with cognition following acute exercise (18, 37). Although we only saw small improvements in affect following the running portion, past research suggests increases in affect can mediate the acute physical activity—executive functioning relationship (38). Differences in our measure of affect [1-item vs. 10-items used in (38)], and/or the type of acute physical activity performed, may have resulted in variations in affect between studies. Nevertheless, future research is encouraged to test sequential mediational models (i.e., physical activity→ changes in affect→ self-efficacy→ changes in executive functioning) to further understand the complex psychophysiological cascade of events following acute physical activity that lead to changes in executive functioning.

### Strengths and Limitations

There were several encouraging and novel findings from the present study, however limitations are notwithstanding. Arguably, the biggest limitation pertained to termination of the study due to the COVID-19 pandemic and this resulted in a relatively small sample size (*N* = 15) and a small number of female participants (*n* = 3) limiting the generalizability of the results, despite attaining consent from 50 potential participants (of which 21 were female). The disruption of data collection also resulted in a lack of randomization at the group level (i.e., counterbalancing the order of the experimental and control conditions) even though this study was originally designed to utilize a counterbalanced within-subject experimental design. Despite the small sample size, we employed a within-subject pre-test post-test experimental design that has been advocated when testing the acute effects of physical activity/exercise on executive functioning (18) and, in turn, strengthened our findings. Although we found a significant effect for inhibition scores, we acknowledge the limited sample size which does not allow us to conclude (i.e., at a level of statistical significance *p* < 0.05) that the program led to changes in switching and updating despite observing small-medium effects for changes in these outcomes. Future research is needed utilizing a greater sample size and an equal number of males and females in order to further test the effects of an afterschool running and reading program on executive functioning among children.

While we were able to control the length of the running portion of the program (i.e., every child participated in 15-min) and RPE scores suggested the participants were working at a “strong” intensity (i.e., a score of 7), the variability in RPE scores suggest some children may have been working too intense (or too light) which may have negatively affected changes in executive functioning. Although the acute physical activity intensity—executive functioning performance relationship remains complex (18), it would be worthwhile in future research to attain measurements of heart rate to help control exercise intensity within each child. The afterschool program leaders delivered the running portion of the study which presented us with ecological validity, however it would be interesting to investigate various types of physical activity (i.e., circuit-based exercise) and/or a combination of types in comparison to running alone on both psychological outcomes (e.g., enjoyment) and cognitive outcomes. Finally, we collected a small amount of demographic information (i.e., only age and gender) which tempers the generalizability of our findings.

## Conclusion

The present study has provided the first evidence for the acute effects of a community-based, program leader delivered, afterschool running and reading program on executive functioning in children. The results from this exploratory study suggest the running portion of the program led to improvements in executive functioning when compared to the sedentary reading portion. However, the study was limited to a small sample size resulting from data collection being ceased due to the COVID-19 pandemic. Thus, further research is needed to elucidate the acute effects of physical activity on executive functioning resulting from this afterschool program.

## Data Availability Statement

The raw data supporting the conclusions of this article will be made available by the authors, without undue reservation.

## Ethics Statement

The studies involving human participants were reviewed and approved by the University of Toronto Research Ethics Board and the Dufferin-Peel Catholic District School Board Research Ethics Committee. Written informed consent to participate in this study was provided by the participants' legal guardian/next of kin.

## Author Contributions

JG conducted the data analyses and drafted the initial manuscript. JG and EB designed the study, coordinated and carried out recruitment, and data collection. CB, PD, and MO assisted in data collection. JC supervised the design and execution of all phases of the study. All co-authors reviewed and approved the final manuscript.

## Conflict of Interest

The authors declare that the research was conducted in the absence of any commercial or financial relationships that could be construed as a potential conflict of interest.

## References

[1] Biddle SJ Ciaccioni S Thomas G Vergeer I Physical activity and mental health in children and adolescents: an updated review of reviews and an analysis of causality. Psychol Sport Exerc. (2019) 42:146–55. 10.1016/j.psychsport.2018.08.011

[2] Poitras VJ Gray CE Borghese MM Carson V Chaput JP Janssen I Systematic review of the relationships between objectively measured physical activity and health indicators in school-aged children and youth. Appl Physiol Nutr Metab. (2016) 41:S197–239. 10.1139/apnm-2015-0663 27306431

[3] Hillman CH Logan NE Shigeta TT A review of acute physical activity effects on brain and cognition in children. Transl J Am College Sports Med. (2019) 4:132–6. 10.1249/TJX.0000000000000101

[4] Sallis JF Bull F Guthold R Heath GW Inoue S Kelly P. Progress in physical activity over the Olympic quadrennium. Lancet. (2016) 388:1325–36. 10.1016/S0140-6736(16)30581-5 27475270

[5] Roberts KC Yao X Carson V Chaput JP Janssen I Tremblay MS. Meeting the Canadian 24-hour movement guidelines for children and youth. Health Rep. (2017) 28:3–7. 29044440

[6] Metcalf B Henley W Wilkin T. Effectiveness of intervention on physical activity of children: systematic review and meta-analysis of controlled trials with objectively measured outcomes (EarlyBird 54). BMJ. (2012) 345:e5888. 10.1136/bmj.e5888 23044984

[7] Pate RR Dowda M. Raising an active and healthy generation: a comprehensive public health initiative. Exerc Sport Sci Rev. (2019) 47:3–14. 10.1249/JES.0000000000000171 30334849

[8] Bedard C St John L Bremer E Graham JD Cairney J. A systematic review and meta-analysis on the effects of physically active classrooms on educational and enjoyment outcomes in school age children. PLoS ONE. (2019) 14:e0218633. 10.1371/journal.pone.0218633 31237913 PMC6592532

[9] Yuksel HS Sahin FN Maksimovic N Drid P Bianco A. School-based intervention programs for preventing obesity and promoting physical activity and fitness: a systematic review. Int J Environ Res Public Health. (2020) 17:347. 10.3390/ijerph17010347 31947891 PMC6981629

[10] Lai SK Costigan SA Morgan PJ Lubans DR Stodden DF Salmon J. Do school-based interventions focusing on physical activity, fitness, or fundamental movement skill competency produce a sustained impact in these outcomes in children and adolescents? A systematic review of follow-up studies. Sports Med. (2014) 44:67–79. 10.1007/s40279-013-0099-9 24122775

[11] Kriemler S Meyer U Martin E van Sluijs EM Andersen LB Martin BW. Effect of school-based interventions on physical activity and fitness in children and adolescents: a review of reviews and systematic update. Br J Sports Med. (2011) 45:923–30. 10.1136/bjsports-2011-090186 21836176 PMC3841814

[12] Demetriou Y Gillison F McKenzie TL After-school physical activity interventions on child and adolescent physical activity and health: a review of reviews. Adv Phys Educ. (2017) 7:191–215. 10.4236/ape.2017.72017

[13] Mears R Jago R. Effectiveness of after-school interventions at increasing moderate-to-vigorous physical activity levels in 5-to 18-year olds: a systematic review and meta-analysis. Br J Sports Med. (2016) 50:1315–24. 10.1136/bjsports-2015-094976 27222308

[14] Kahan D McKenzie TL Physical activity and psychological correlates during an after-school running club. Am J Health Educ. (2018) 49:113–23. 10.1080/19325037.2017.1414646 29399835

[15] Scott-Andrews KQ Cosgrove JM Robinson LE Castelli DM Improving adolescent health: a comparison of 2 after-school programs. Health Behav Policy Rev. (2020) 7:92–101. 10.14485/HBPR.7.2.2

[16] Durlak JA Weissberg RP Pachan M. A meta-analysis of after-school programs that seek to promote personal and social skills in children and adolescents. Am J Commun Psychol. (2010) 45:294–309. 10.1007/s10464-010-9300-6 20300825

[17] Faul F Erdfelder E Buchner A Lang AG. Statistical power analyses using G^*^Power 3.1: Tests for correlation and regression analyses. Behav Res Methods. (2009) 41:1149–60. 10.3758/BRM.41.4.1149 19897823

[18] Pontifex MB McGowan AL Chandler MC Gwizdala KL Parks AC Fenn K A primer on investigating the after effects of acute bouts of physical activity on cognition. Psychol Sport Exerc. (2019) 40:1–22. 10.1016/j.psychsport.2018.08.015

[19] Miyake A Friedman NP Emerson MJ Witzki AH Howerter A Wager TD. The unity and diversity of executive functions and their contributions to complex “frontal lobe” tasks: a latent variable analysis. Cogn Psychol. (2000) 41:49–100. 10.1006/cogp.1999.0734 10945922

[20] Wade L Leahy A Lubans DR Smith JJ Duncan MJ. A systematic review of cognitive assessment in physical activity research involving children and adolescents. J Sci Med Sport. (2020) 23:740–5. 10.1016/j.jsams.2019.12.020 31911043

[21] Stroop JR Studies of interference in serial verbal reactions. J Exp Psychol. (1935) 18:643 10.1037/h0054651

[22] Reitan RM. Validity of the Trail Making Test as an indicator of organic brain damage. Percept Motor Skills. (1958) 8:271–6. 10.2466/pms.1958.8.3.27115010091

[23] Lezak MD Howieson DB Loring DW Neuropsychological Assessment. 4th ed New York, NY: Oxford University Press (2004).

[24] Roid GH Miller LJ Pomplun M Koch C. Leiter International Performance Scale. 3rd ed. Wood Dale, IL: Stoelting Company (2013).

[25] Bandura A Self-Efficacy: The Exercise of Control. New York, NY: Freeman (1997).

[26] Beauchamp MR Crawford KL Jackson B Social cognitive theory and physical activity: mechanisms of behavior change, critique, and legacy. Psychol Sport Exerc. (2019) 42:110–7. 10.1016/j.psychsport.2018.11.009

[27] Ekkekakis P Parfitt G Petruzzello SJ. The pleasure and displeasure people feel when they exercise at different intensities. Sports Med. (2011) 41:641–71. 10.2165/11590680-000000000-00000 21780850

[28] Teixeira PJ Carraça EV Markland D Silva MN Ryan RM. Exercise, physical activity, and self-determination theory: a systematic review. Int J Behav Nutr Phys Activ. (2012) 9:78. 10.1186/1479-5868-9-78 22726453 PMC3441783

[29] Deci EL Ryan RM The “what” and “why” of goal pursuits: human needs and the self-determination of behavior. Psychol Inquiry. (2000) 11:227–68. 10.1207/S15327965PLI1104_01

[30] Ryan RM Control and information in the intrapersonal sphere: An extension of cognitive evaluation theory. J Personal Soc Psychol. (1982) 43:450 10.1037/0022-3514.43.3.450

[31] Bandura A Guide for constructing self-efficacy scales. In: Pajares F Urdan TC editors. Self-efficacy Beliefs of Adolescents. Greenwich, CT: Information Age Publishing (2006). p. 307–37.

[32] Hardy CJ Rejeski WJ. Not what, but how one feels: the measurement of affect during exercise. J Sport Exerc Psychol. (1989) 11:304–17. 10.1123/jsep.11.3.30418835411

[33] Borg G Borg's Perceived Exertion and Pain Scales. Champaign, IL: Human kinetics (1998).

[34] IBM Corp. IBM SPSS Statistics for Macintosh, Version 25.0. Armonk, NY: IBM Corp (2017).

[35] Cohen J. A power primer. Psychol Bull. (1992) 112:155. 10.1037/0033-2909.112.1.155 19565683

[36] Hillman CH Biggan JR. A review of childhood physical activity, brain, and cognition: perspectives on the future. Pediatr Exerc Sci. (2017) 29:170–6. 10.1123/pes.2016-0125 27615274

[37] Hillman CH Kamijo K Pontifex MB The relation of ERP indices of exercise to brain health and cognition. In: Boecker H Hillman CH Scheef L Strüder KH editors. Functional Neuroimaging in Exercise and Sport Sciences. New York, NY: Springer (2012). p. 419–46.

[38] Schmidt M Benzing V Kamer M. Classroom-based physical activity breaks and children's attention: cognitive engagement works! Front Psychol. (2016) 7:1474. 10.3389/fpsyg.2016.01474 27757088 PMC5047899

